# Supplementary material for: Are carbon emissions trading and green financial instruments synergistic? -Comprehensive quantitative research based on content analysis
Source: PLoS One. 2024 Mar 7;19(3):e0298601. doi: 10.1371/journal.pone.0298601 (PMC10919601; doi:10.1371/journal.pone.0298601)
Supplement: S1 Data — (ZIP) [file pone.0298601.s001.zip › Data/地级市Z_F工作报告.zip!/║■─╧/╙└╓▌╩╨/└┤╫╘╣1⁄2╓┌║┼í╛┬φ┐╦╩2╛▌═°í┐_2014.docx]

各位代表：

现在，我代表市人民政府向大会报告工作，请予审议，并请各位政协委员和其他列席人员提出意见。

一、2013年工作回顾

过去的一年，是我市在积极探索中奋力前行、在困难挑战中稳步发展的一年。一年来，面对经济下行压力加大、前旱后涝灾害叠加带来的不利影响，全市上下在市委的坚强领导下，迎难而进，奋发有为，经济社会发展取得实效，改革创新能量加速聚集。

经济发展稳中有进。全年地方生产总值完成1163．4亿元（预计数，下同）、增长10％，固定资产投资突破千亿，达到1071．9亿元、增长33．5％，社会消费品零售总额完成374．4亿元、增长13．7％，财政收入超过百亿，达到100．4亿元、增长15．8％，城乡居民收入达到22368元、7799元，分别增长12．5％和13％。工业经济企稳回升。规模工业增加值完成260亿元、增长12％，规模工业企业新增90家、总数达到737家，高新技术企业新增6家、总数达到47家。零陵烟厂、猎豹汽车支撑作用继续加强。电子信息、新能源新材料、生物医药等新兴产业来势较好，冶炼、建材、造纸等传统产业整合提升，全年淘汰铁合金等落后产能11．8万吨、水泥36万吨，关闭“五小”企业65家。工业固定资产投资、技术改造投入分别完成432．4亿元和220亿元。农业形势总体平稳。粮食、蔬菜产量因灾有所下降，其它农产品生产稳中有增，果茶扩园6．6万亩、低改29万亩，8家蔬菜基地创建为国家级蔬菜标准园，祁阳被评为全国粮食生产先进县，江永成为全国绿色食品原料标准化生产基地。烤烟收购72．1万担，产业效益创历史最好水平。畜牧水产养殖平稳增长，新建市级标准化规模养殖场100个、省部级标准化规模养殖场11个。农业产业化持续推进，新增市级以上龙头企业11家、总数达到214家。农业服务体系不断健全，新发展农民合作社348个、农超对接连锁店202家、“网上供销社”乡村服务站点201个。农村土地流转扎实开展，新增流转面积53万亩。农业机械化稳步推进，新增各类机械5．1万台套，水稻机插面积突破100万亩。现代农业示范园区创建工程取得实效，永州国家农业科技园建设初具规模。第三产业持续提升。旅游业保持较快发展，零陵古城、阳明山、九嶷山、金洞等旅游项目建设进展顺利，柳子景区成功创建4A景区。汽车、家电、住房等消费热点持续升温，电子商务、现代物流等新型业态逐步发展。金融保险服务功能增强，全市金融机构存款余额达到1172．6亿元、增长17．3％，贷款余额达到554．4亿元、增长19．7％，保费收入完成32亿元、增长33％。归集住房公积金12．6亿元。蓝山、祁阳农村信用社改制为农村商业银行，交通银行、华融湘江银行进驻永州，新增小额贷款公司7家、总数达到11家，市城建投二期10亿元债券成功发行。县域经济发展提速。祁阳跻身全省首批特色县域经济重点县，冷水滩、江华被评为全省县域经济发展先进县，祁阳、宁远被评为全省发展非公有制经济和中小企业先进县。各县区都有一批特色亮点工作进入国家和省里先进行列。

城乡建设展现新貌。交通发展实现新突破。湘桂高速铁路全面开通，结束了永州没有高铁的历史，标志着永州跨入高铁时代。永州至深圳航线即将开通。干线公路建设扎实推进，完成新改建295公里，二广高速双牌连接线、省道325道县祥霖铺至江永龙虎关、国道207东安段和江华段、国道322黄母桥至枣木铺改造工程全面完成，祁冷一级公路路基全线拉通。农村公路改造完成701公里，危桥改造完成100座，客运站场建成2个，农村客运招呼站建成180个。湘江千吨级航道衡阳松柏至永州蘋岛项目获批，衡道高速公路、永州机场搬迁等前期工作抓紧进行。城镇面貌发生新变化。中心城市启动城建重点项目141个，完成投资56．4亿元，湘江风光带、城南大道、湘江东路、湘江西路建设进展较快，零陵新区“四纵四横”路网骨架全面拉开，城区提质工作扎实推进，完成背街小巷改造40条，打通断头路5条，新改建农贸市场57家，中心城区面貌有了很大改观。县城和中心镇建设成效明显，江华通过全国文明县城复查验收，宁远列为全省新型城镇化示范县，东安、新田创建为省级卫生县城。农业基础建设取得新进展。涔天河水库扩建工程扎实推进，灌区及耕地后备资源开发项目获批。201座小二型水库除险加固主体工程基本完工，4个全国小农水重点县和11个中小河流治理项目开工建设，水毁工程和“五小”水利工程完成2．04万处，新解决34万农村人口饮水安全问题。完成中低产田改造和高标准农田建设11．8万亩，实施土地综合整治及土地开发24．2万亩，新增耕地3．7万亩。道县跻身全国建设高标准基本农田示范县，东安、江华、道县、宁远被评为全省节约集约用地模范县。省定为民办实事新农村示范村、“百城千镇万村”新农村建设工程和“乡村风貌”示范片建设扎实推进。生态文明建设达到新水平。全市完成造林72．88万亩，中心城区大苗种植20．6万株，我市被确定为全国油茶产业发展示范市。节能减排任务全面完成，湘江治理保护“三年行动计划”进展顺利。双牌被列为国家绿色能源示范县，江华被列为全国绿化模范县，九嶷山、舜皇山创建国家级自然保护区，涔天河湿地公园获批全市首个国家湿地公园，双牌日月湖湿地公园通过国家评审。

示范区建设扎实推进。招商引资成效明显。全年实际利用外资8亿美元、增长27．4％，到位内资465亿元、增长17．4％；落户承接产业转移项目400个，其中亿元项目105个。我市正逐步成为具有区域影响力的轻纺制鞋、电子信息、新能源新材料产业制造基地。对外贸易不断扩大。外贸进出口总额完成3．9亿美元、增长35％，其中加工贸易进出口总额完成2．2亿美元、增长57％。新增有进出口实绩的企业14家、总数达到64家，进出口总额过1000万美元的企业达到10家。园区建设得到加强。工业园区新开发面积32平方公里，新建标准厂房213万平方米，新入园企业160家。永州经济技术开发区申报国家级经开区进展顺利，道县工业园获批全省首批新能源产业园，零陵锰产业专业园列入全省新型工业化示范基地，双牌、江永工业园获批省级工业集中区。

民生事业全面进步。全年完成各项民生支出185亿元、增长22．2％，占全市财政总支出的72．5％。社会保障不断完善。新增城镇就业6．1万人、农村劳动力转移就业6．1万人，帮扶0．8万就业困难人员实现再就业，307户零就业家庭实现至少1人就业。“五项保险”参保人数达到638万人次。企业退休人员养老金、城乡低保月人均救助、五保老人分散供养标准继续提高。在全省率先启动城乡居民大病保险，新农合参合率达到98．9％。新建保障性住房1．29万套，实施农村危房改造1．17万户，完成因灾倒房重建2003户。省定为民办实事任务全面完成。社会事业协调发展。新建农村合格学校73所、农村公办幼儿园27所、农村教师周转房1330套，新增城区学位1．63万个，其中中心城区4530个，城区大班额问题有所缓解。永州综合职业中专入围省示范性中职学校，永州工商职业中专通过国家改革发展示范校评估验收。实施国家级科技计划项目6项、省级科技计划项目54项，授予专利606件，我市跻身国家知识产权试点城市，祁阳、冷水滩、东安被评为全国科技进步先进县，东安被评为全国知识产权试点强县。医疗卫生事业稳步发展，市妇幼保健院、儿童医疗服务中心等项目建设进展顺利，基本医药制度覆盖面持续扩大，重大疾病防控和卫生应急能力有新提升，全市未发生重特大食品药品安全事故。文化惠民工程深入实施，公共文化服务体系不断完善，新增19处全国重点文物保护单位。群众体育蓬勃开展，公共体育设施建设不断加强，江华被评为全国群众体育先进县。人口计生工作实现保先进位。扶贫、移民开发有新成效，全年减少贫困人口13万，完成移民帮扶7．9万人。工青妇等群团组织作用充分发挥。“六五”普法扎实推进。中国特色社会主义和“中国梦”宣传教育深入开展，精神文明创建活动不断深入。统计、审计、质监、物价、工商、民族宗教、外事侨务、贸促、广电、对台、史志、档案、气象、水文、人防、消防、残联、老龄、慈善、红十字会等工作都有新的进步。社会大局和谐稳定。安全生产工作保持全省先进，各类事故起数、死亡人数同比分别下降61％和31％。信访维稳工作全面加强，平安城市、智慧公安、阳光警务建设扎实推进，“两抢一盗”等多发性侵财案件大幅下降，现行命案侦破率排全省第一，综治民调工作继续在全省领先。

依法行政不断加强。牢固树立法制意识，严格执行市人大及其常委会的决议、决定，自觉接受市人大及其常委会的法律监督和工作监督，主动接受市政协的民主监督，广泛听取各民主党派、工商联、无党派人士的意见建议。办理市人大代表议案、建议187件，市政协委员提案225件，办复率达到100％。大力推进简政放权，市本级保留行政审批项目166项，审批时限在法定时间内缩短“两个1/3”以上。率先在全省实行建设工程项目并联审批，积极推进网上政务服务和电子监察系统应用，市公共资源交易中心成立并启用，市、县两级“12345”公共服务平台全面开通。扎实推进绩效考核，严格规范部门执法，切实强化行政问责，政府公信力和执行力进一步增强。

国防和后备力量建设成效明显。规范人民武装委员会和国防动员机构设置，后备力量建设和保障更加规范化、制度化。全民国防教育深入推进，双拥共建和“双带双促”氛围更加浓厚。民兵和征兵工作改革顺利推进，优抚安置政策更加完善。驻永解放军、武警部队和民兵预备役人员积极参建参治，为全市经济社会发展做出了重要贡献。

一年来，我们按照市委决策部署，着重围绕以下四个方面开展工作：

一是谋大事。坚持思路不改、目标不变、力度不减，着眼长远想大事、谋全局。围绕突破“中心城市、新型工业化、基础设施”三大薄弱环节，在提升中心城市承载力上，明确了扩容提质的总体思路，制定了三年提质计划，理顺了市区管理体制；在推进新型工业化上，出台了引进培植壮大骨干企业的政策措施，深化了工业企业服务年活动；在完善基础设施配套上，加快了交通、园区、物流、电力等平台建设，进一步强化了发展支撑。围绕增强农村发展活力，确定了推进农村土地流转制度改革的目标任务和工作重点。围绕加强招商引资工作，组织开展小分队招商，对2010年以来签约的近400个招商项目履约情况进行了集中清理、分类处置。这些措施的制定出台，为全市的长远发展提供了坚实的政策支撑。

二是抓关键。我们坚持以项目建设统揽发展全局，突出以项目实绩评定干部政绩。加快推进“十百千工程”项目建设，猎豹汽车、零陵烟厂、希尔制药、冷水滩德信泰和、祁阳凯迪生物质发电、江华风电、零陵瑞祥新材料、宁远亿达、道县美莱铂、蓝山必达电子、东安格润新能源、江永荣旺食品、双牌华瑞科技、新田家俬产业园等一批产业项目，湘桂高铁、国省干线公路改造、老山界220千伏变电站扩建等一批基础设施项目，合格学校建设、医疗卫生服务体系建设、农村安全饮水等一批民生项目相继建成，神华国华永州火电、湘纸异地技改、洛湛铁路复线等一批重大项目前期工作取得实效。建立健全领导联系制度、联席会议制度、定期调度制度，强化责任落实，严格奖惩兑现。开展比学赶超，年内两次召开全市重点项目流动现场会，全市上下形成了赶先进、争上游、创一流的浓厚氛围。

三是解难题。针对经济社会发展中存在的突出问题，勇于直面、敢于担当，创新方式、寻求突破。在面对特大旱涝灾害上，全市上下全力以赴，共克时艰，取得了抗灾救灾和灾后建设的胜利，把灾害损失降到了最低程度；在破解融资难题上，制定出台了促进金融业加快发展的意见，整合资源、完善体制，组建市城建集团和市经建集团。在破解征地拆迁难题上，研究出台新的征地拆迁安置补偿办法，鼓励和引导货币安置；在处理中心城区历史遗留问题上，强力推进控违拆违，全年拆除违法建筑54万平方米，集中办理小区房产、国土两证，103个商品房小区已办理房产证40771户，办理土地使用证26716户；在方便市民出行上，中心城区新增公交营运线路4条，更新车辆300台，新增出租车200台。

四是促落实。加强政府廉政建设和机关效能建设，严格执行中央“八项规定”、省委“九条规定”和市委“十项规定”，坚决反对和抵制“四风”，以良好的政风带民风树正风。扎实开展“转作风、解难题、抓关键、见实效”专项活动，深入基层、深入群众，排忧解难，帮扶解困。始终坚持以责任为“镜子”、以监督为“尺子”、以奖惩为“鞭子”，严格督促政府部门及其工作人员一门心思干工作，扑下身子抓落实。

各位代表，过去的一年，我们能够取得这些成绩，得益于省委、省政府的高度重视，得益于市委的科学决策，得益于市人大、市政协的监督支持，得益于全市上下的不懈努力。在此，我代表市人民政府，向全市人民，向驻永部队、武警官兵，向所有关心支持永州建设发展的各级领导、各界朋友，表示衷心的感谢，并致以崇高的敬意！

在肯定成绩的同时，我们也清醒认识到，工作中仍存在不少困难和问题，主要是：经济发展速度有所放缓，地方生产总值、规模工业增加值增幅与年初目标有较大差距；新型工业化、中心城市、基础设施仍然薄弱，全面小康建设任务艰巨；社会保障水平不高，部分群众生活比较困难；“三强”行为没有得到有效遏制，建设环境仍然不优；少数政府工作人员法制意识不强，不作为、慢作为、乱作为等现象仍然存在。对此，我们一定高度重视，在今后的工作中认真加以解决。

二、2014年工作安排

2014年是贯彻落实党的十八届三中全会精神、全面深化改革的第一年，也是为全面建成小康社会奠定坚实基础的一年。新的一年，机遇条件仍然较多，但外部环境错综复杂。党的十八届三中全会作出了全面深化改革的战略部署，随着各项改革的深入推进，发展的强大动力、内需的巨大潜力将不断释放。我省充分发挥“一带一部”的区位优势，建设“四化两型”，促进“三量齐升”，守住“四条底线”，新一轮发展热潮正在形成。但必须看到，世界经济虽然延续缓慢复苏态势，但发达经济体量化宽松政策调整、新兴市场国家增速减缓的影响难以估量，不稳定不确定因素仍然较多。国内经济稳中向好的基础还不稳固，经济下行压力依然存在，稳中有忧、稳中有险。新的一年，发展基础比较扎实，但建设任务十分繁重。近年来，在历届市委、市政府的正确领导下，全市经济社会发展较快、成效明显，但必须看到，我市人口多、底子薄、欠发达等三大基本市情还没有根本改变，新型工业化、中心城市、基础设施等三大薄弱环节还没有根本突破，经济总量、人均均量、发展质量等三大经济指标还没有根本提升，加快发展始终是永州压倒一切的首要任务，任何时候都不能动摇、不可松懈。新的一年，内生动力持续增强，但竞争态势相当紧迫。当前支撑我市加快发展的利好因素较多，涔天河水库扩建、神华国华永州火电等一批重大项目加快建设，零陵烟厂、猎豹汽车等骨干企业形势向好，县域经济活力进一步增强。但必须看到，全国各地抓住全面深化改革的有利时机，不断激发市场活力、提升发展水平；我省长株潭城市群、环洞庭湖区域抢抓政策机遇，已站在更高的发展起点，大湘西地区利用武陵山片区扶贫开发政策，兴起了新一轮开发建设热潮，同属湘南板块的衡阳、郴州，同处“千亿俱乐部”的兄弟市州发展势头强劲。全市上下一定要增强忧患意识、树立底线思维，坚定发展信心、抢抓改革机遇，努力迈出科学发展、加快赶超步伐。

工作总体要求是：全面贯彻落实党的十八大、十八届三中全会和中央、省委、市委经济工作会议精神，坚持以经济建设为中心，以加快科学发展为第一要务，以分类指导全面建成小康社会为总揽，牢牢把握“稳中求进、改革创新”核心要求，突出稳增长、调结构、促改革、打基础、惠民生，促进“三量齐升”，推进“四化两型”，切实提高经济发展质量和效益，加快建设品质活力永州，为富民强市、赶超崛起奠定坚实基础。在工作重点上要着力实现新型工业化、中心城市建设、基础设施建设三大突破，推进行政管理体制、农村产权制度、社会治理机制三项改革，坚持生态、教育、就业三项优先，抓好城镇与农村、当前与长远、经济与社会发展三项统筹。

主要预期目标是：地方生产总值增长10．5％左右，规模工业增加值增长12％，固定资产投资增长25％，社会消费品零售总额增长14％，财政总收入增长15％，城乡居民收入增长13％。全面小康实现程度提高2．5个百分点左右。人口自然增长率控制在9．2‰以内。坚决完成资源节约、环境保护、转型升级、安全生产等约束性指标。

工作中必须牢牢把握五个方面：

坚持以制度创新推动跨越赶超。加快制度创新是全面深化改革的重要内容和关键环节，要以思想解放的新跨越带动制度创新的大突破，通过制度创新推动管理转变、完善公共服务、优化发展环境，以改革创新构筑制度新高地、提升发展“软实力”。

坚持以“高铁”思维谋划工作全局。湘桂高铁全面开通，为我市承接产业转移、推动转型升级、融入区域发展带来了难得的历史机遇，要打破思维定势、行为惯性和路径依赖，运用“高铁”思维，放大“高铁”效应，以更加超前的眼光、更加开放的心态、更加高效的工作谋划和推动全市发展。

坚持以项目建设扩大有效需求。项目建设始终是我市加快发展的第一推动力，要以项目建设为统揽，以“十百千工程”为重点，力抓项目，快上项目，早成项目，在全市上下营造齐心协力干项目、全力以赴促发展的浓厚氛围。

坚持以问题导向突破瓶颈制约。问题导向是人类文明进步的重要推动力。要把善于发现问题、切实解决问题作为政府工作的基本要求，紧紧抓住事关发展全局的根本问题、社会普遍关注的热点问题、群众反映强烈的突出问题，一个难点一个难点突破，一个问题一个问题解决，抓一件成一件，积小胜为大胜。

坚持以民生优先践行发展宗旨。群众需要是推动政府工作的根本动力，人民满意是检验政府实绩的最高标准。要尽心竭力促发展、保民生，努力让人民群众拥有更好的教育、更稳定的工作、更满意的收入、更可靠的社会保障、更高水平的医疗保健、更舒适的居住条件、更优美的工作环境。

围绕上述思路和目标，突出抓好“八大建设”：

1、以新型工业化为重点，加快推进产业建设

壮大工业经济实力。着力强龙头。全面落实引进培植壮大骨干企业的政策措施，年内新培育亿元企业30家，新入统规模企业100家。充分发挥三大标志性企业的支撑作用，加快猎豹汽车、零陵烟厂技改扩能，实施湘纸异地技改工程。全力推进项目建设，重点抓好神华国华永州火电、台湾高科技产业园、康都制药、湘器异地搬迁、烟草产业园、富士康配套产业园、江华稀土、零陵百亿锰产业、达福鑫、祥瑞科技等一批重大项目。依托骨干企业，延伸产业链条，加强产业配套，促进小微企业发展，形成全民创业热潮，全年新培育小微企业1200家以上。着力兴园区。坚持把园区建设作为工业发展的主战场，按照“工业新城、城市新区”的理念，全面提升产业集聚、功能集成水平，全年工业园区完成技工贸总收入1100亿元，建成标准厂房200万平方米。永州经济技术开发区要加快“一区三园”融园步伐，力争年内成功申报国家级经开区，蓝宁道新加工贸易走廊要打造成全省第一家加工贸易特色区。推进县区工业园“五个一”建设，全年每个园区引进落户规模工业企业10家以上、基础设施投入1亿元以上、扩园面积1平方公里以上、土地储备1000亩以上、新建标准厂房10万平方米以上。创新园区建设方式，积极与沿海城市、企业、东盟国家合作共建产业园区。着力促转型。培育新兴产业促转型，重点发展先进装备制造、电子信息、新能源新材料、生物医药产业，启动实施两化融合“111”工程。提升传统产业促转型，充分利用国家政策导向和市场倒逼机制，加快农产品加工、矿产品加工、轻纺制鞋等传统产业的改造提升，坚决淘汰落后设备和过剩产能，培育传统产业新的竞争优势，整合资源，着力打造千亿矿业经济带。积极盘活存量促转型，加快国有企业改革扫尾，对尚未改制的28家市属国有企业，逐个制定方案、落实措施，力争年内改制到位。

增创农业发展优势。突出抓好五大工程建设。一是主导产业提升工程。大力实施农产品加工业振兴计划，着力推动集群发展，新培育年销售收入过亿元企业3家。围绕粮油、畜禽、烤烟、果蔬、林木等优势产业，建设400万亩优质稻、20万亩高产油茶、50万亩外销蔬菜、100万亩柑橘、50万亩时令水果、26万亩烤烟、2万亩花卉苗木和8万亩有机茶叶等规模生产基地，新创建100个市级畜禽水产养殖标准化示范场。二是名优品牌创建工程。大力推进农业标准化，完善标准体系，抓好标准示范，着力打造品牌农业、绿色农业和休闲观光农业。宣传推介江永“五香”、新田富硒、道州脐橙、廻峰柑橘、永州异蛇、东安鸡、九嶷山兔等优质农产品，切实提高对外知名度和市场影响力。鼓励农业龙头企业创建著名品牌和驰名商标，全年新培育3个省级以上名牌产品。加快永州国家农业科技园建设，新引进重大产业项目10个以上。三是服务体系完善工程。加强农业科技创新和推广应用，健全农业综合服务体系。提高农机化水平，建设全程机械化作业高产示范基地。加大农业信贷投入，健全政策性和商业性农业保险制度。抓好防汛抗旱、森林防火和重大动物疫病防控工作。切实加强农产品生产源头治理和产销全程监管，严厉打击食品安全犯罪，确保人民群众“舌尖上的安全”。四是新农村建设工程。继续推进“乡村风貌”示范片、省定为民办实事新农村示范村等建设，每个县区要成片推进好3个以上示范村建设。统筹编制好以城带乡的各类专项规划，全年每个县区完成5个以上行政村的规划编制。五是新型农业经营主体培育工程。建立健全新型农业经营主体，大力发展家庭农场、专业大户、农民合作社、产业化龙头企业，全年新发展农民合作社200个。培养造就新型农民队伍，把培养青年农民纳入全市实用人才培养计划，积极培育发展职业农民队伍。

提升第三产业水平。促进现代服务业提质发展。制定现代物流业发展规划，促进现代物流、研发设计、服务外包等生产性服务业发展，重点抓好城市综合体等项目建设，培育发展一批年交易额过10亿元的商圈、年营业额过1亿元的商贸物流企业。抓好国家级农产品现代流通综合试点城市工作，实施农贸市场改造提升工程，扩大农超对接和直供直销规模。大力发展社区、家政、养老、健康等生活性服务业，拓展文化、体育健身、娱乐、信息等新兴服务消费，积极发展电子商务、网络购物、电视购物等新型流通业态。促进旅游业加快发展。大力促进文化旅游深度融合，倾力打造零陵古城和阳明山片区“古城名山”核心品牌，以创建国家历史文化名城为抓手，彰显古城风貌，以潇湘平湖为依托，打造水陆联动的景观长廊，以建设国家对台交流基地为契机，抓好阳明山景区的提质改造。加快实施浯溪碑林、九嶷山、舜皇山、江永“三千”文化园、蓝山湘江源头保护开发、江华瑶族文化园、道县濂溪文化产业园、新田孝文化公园等精品工程。启动实施市旅游集散中心建设。推进九嶷山景区创5A工作。加强名镇、名居、名胜保护，延续历史文脉，记住故园乡愁，彰显地域特色。促进房地产业稳步发展。合理引导住房需求，完善住房供应体系，加强中小户型、中低价位普通商品住房建设。加强保障性住房建设，推进棚户区改造。规范房地产市场秩序，优化房地产发展环境。促进金融业创新发展。扩大有效信贷投入，全年新增贷款120亿元。构建新型农村金融体系，争取2家农村信用社改制为农村商业银行，新引进1家股份制商业银行，新组建1家村镇银行，实现小额贷款公司和融资性担保机构县区全覆盖。年内新增3家省重点上市后备企业，争取1－2家企业上市。积极做好企业债发行准备工作。支持市城建集团和市经建集团做实做强。依法扩大住房公积金覆盖面，着力推进非公有制经济建立公积金制度。全面推进社会信用体系建设，深入开展金融安全区创建，进一步优化金融生态环境。

2、以打造品质活力城市为重点，加快推进城镇建设

着力完善城镇规划。明确城市发展定位，坚持以人为本、着眼未来发展，努力把永州建设成为品质活力城市，全面提升城市的文化品质、生态品质、建设品质、产业品质、生活品质，让各类资源要素活力竞相迸发、充分涌流。优化城镇空间布局，积极开展中心城市各片区开发、旧城改造以及重大项目策划，把中心城市扩容与祁阳、东安、双牌县城发展统筹考虑，抓好规划衔接，作为潇湘城市群进行整体谋划。完善城镇规划编制，深化中心城市总体规划，完成片区控规修改和专项规划编制；扎实推进零陵历史文化名城、永州经济技术开发区和滨江新城控规修编；启动高溪市镇、上岭桥镇、伊塘镇和蔡市镇总体规划编制；按照统筹城乡发展的要求，编制完善城镇体系规划、村镇规划。

扎实推进城镇扩容。充分发挥市城建集团和经建集团两大平台作用，分片区推进中心城市建设，年内抓好湘江东路、湘江西路、城南大道、潇湘大道、城南大桥、阳明大道、湘江风光带等项目建设，启动实施规划馆、文化艺术中心、体育中心等项目。县城扩容要依托产业园区、功能新区建设，拉开路网骨架，扩大城镇规模，结合区位、交通、资源等要素，引导小城镇发展特色产业，着力打造一批文化旅游名镇、工业商贸重镇，每个县区重点抓好1个以上特色示范镇建设。按照既要坚持依法依规、又要考虑群众利益的原则，做好征地拆迁工作，中心城区要积极实施货币补偿、产权置换、公寓楼安置三种新的安置办法。

切实提升城镇品质。以创建国家卫生城市和国家历史文化名城为抓手，全面实施中心城市三年提质计划。加快创国卫步伐，确保3月底前省里同意申报，争取尽快通过国家评审。完善城市服务功能，全面推进商业网点、文化设施、医疗卫生设施、停车场、地下管网、给排水等功能配套，大力发展绿色公共交通，坚持公车公营，中心城区要抓好零陵路、湘江东路提质和梅湾路、南津路建筑立面改造，开工建设零陵中路单建式人防工程，打通19条断头路，完成98条背街小巷改造，抓好曲河等5个自来水厂扩容配套，以及下河线、向家亭和马坪污水处理设施扩建，完成14个安置小区建设。强化城市管理，全面启动数字城管建设，切实提高城市网格化、精细化管理水平，大力开展控违拆违、交通秩序等集中整治行动，突出抓好建设工地环境治理和渣土车、施工车管理。积极开展文明县城、卫生县城、园林县城等创建活动，提升城镇建设和管理水平。

3、以交通建设为重点，加快推进基础设施建设

完善交通格局。抢抓国家加快中西部铁路建设机遇，全力推动洛湛铁路益永段、湘桂铁路高溪市镇至永州站新建三线前期工作，争取贵永郴、桂永郴赣铁路列入国家“十三五”铁路网规划，积极促成湘桂高铁开通到上海、广州班次。全力推动衡道高速、永郴高速尽早开工，尽快打通二广、厦蓉、道贺三条高速公路外出通道。加快出省跨境公路、干线公路、县与县之间断头路建设，建成祁冷一级公路、国道322零陵段改线工程、厦蓉高速宁远互通至九嶷山连接线、省道355江华白芒营至广西福利公路，加快207国道改造步伐，开工建设冷水滩至东安一级公路、省道348祁阳黎家坪至冷水滩马坪、省道238东安芦洪市至小江口、省道236中和至宁远县城、省道228新田县城至竹林坪、省道355江华分水岭至白芒营、省道239广西灌阳边界至江永潇浦公路，同时加强农村通畅工程建设和农村公路维护管理。积极做好湘桂运河规划立项前期工作，开工建设湘江千吨级航道改扩建工程和3个千吨级港口码头。推动永州机场搬迁工作取得阶段性成果。

改善农田水利。切实加强水利建设，全力推进涔天河水库扩建，做好移民安置工作，加快实施枢纽工程计划。完成63座小二型水库扫尾，抓好凤仙桥、半山、杨家洞等3座中型水库除险加固，推进芦江、金钩挂等水库建设，加快水毁水利工程修复、中型灌区维修改造和沟渠疏浚。完成10个中小河流治理项目和7个全国小农水重点县年度建设任务，争取5个县区列入全省山洪沟治理计划。推进国土整理，加强水土保持和农业综合开发。解决30万农村人口安全饮水问题。

强化能源保障。全力抓好神华国华永州火电项目建设，争取一期主体工程早日开工。加快江华、双牌、江永等地风能开发，推进祁阳生物质能源、东安光伏发电等项目建设。完善电网布局，开工建设新田220千伏输变电工程和宁远城南、东安芦洪市2个110千伏输变电工程。推进中心城市管道燃气项目建设。

打造数字平台。实施《数字永州规划》，重点建设全市云计算中心、数据中心和电子政务网络平台，整合信息资源，实现数据共享。大力发展物联网，加快“三网”融合，推进广播电视“村村通”、宽带“村村通”、城市光网、4G移动通信网络、“网上供销社”等建设。

4、以开放承接为重点，加快推进示范区建设

加快产业承接。全年实际利用外资9亿美元，内联引资500亿元以上，新承接产业转移项目500个以上。突出小分队招商，全年每个市直招商小分队至少要引进1个投资5000万元以上工业或商贸物流项目。突出大企业招商，鼓励市内骨干企业扩大对外合作，开展自主招商。突出产业链招商，围绕先进装备制造、电子信息、新能源新材料、农产品精深加工、矿产品精深加工、生物医药、轻纺制鞋等主导产业，大力承接一批产业领军企业和链条配套企业。突出招商实效，提高合同履约率和资金到位率。

扩大对外贸易。引进一至两家大型进出口代理公司，做大做强外贸主体。鼓励外贸企业申报国际知名品牌，提升出口竞争实力。引导市内企业利用中博会、东博会等展会平台，进一步开拓市场。扩大与东盟国家的经贸往来，帮助恒远发电、果秀食品等企业拓展东盟市场。

深化对接合作。建立与珠三角、长三角、闽三角等重点产业转出地政府、商会、企业的互动渠道和机制，开展项目投资和经贸合作。抓住国家打造升级版的中国东盟自贸区这一战略机遇，利用湘桂高铁全面开通的有利条件，深化与东盟的对接合作，把永州打造成为全省对接东盟的桥头堡。加强湘南三市的交流合作，促进产业建设互补互利、基础设施互联互通。

强化平台支撑。抓好永州无水港建设，确保年底前建成开港。加快建设湖南农副产品集中验放场，构筑全省供港澳及进出口东盟的农产品集中交易、集中验放新平台。积极创造条件，申报设立永州海关和永州出入境检验检疫局。

5、以深化改革为重点，加快推进体制机制建设

切实抓好行政管理体制改革。大力简政放权，减少前置审批，全年市本级再削减和下放审批项目15％以上，部分行政审批项目改为备案事项，把工作重点放在事中事后监管上。优化审批流程，压缩审批时限，全面推行政府投资项目和建设工程项目并联审批制度，继续实施审批提速“两个1/3”工程。改革工商注册制度，全面推行负面清单管理，实行“先照后证”、“宽进严管”，除法律规定外，取消最低注册资本限制，改注册资本实缴制为认缴制。大力推行一站式服务制、一口收费制和检查报备制。规范各级政务中心窗口单位的办理标准和模式，所有审批项目统一纳入全省网上审批和电子监察系统。高标准建设好中小企业服务中心、企业证照办理中心和乡镇便民服务中心。推进公共服务社会化、市场化，实行政府向社会力量购买公共服务。

切实抓好农村产权制度改革。抓好农村集体土地确权工作，上半年耕地确权发证到组，年底前林地和耕地使用权确权基本到户。全面编制农地规划，今年着手组织，明年完成市、县、乡镇三级农用土地利用规划编制。加强流转平台建设，上半年完成市、县、乡镇、村四级土地流转服务网络体系和县、乡、村三级土地流转纠纷调处机构建设，下半年组建市级统一的土地招商平台。按照先试点、后推开的原则，建立城乡统一的建设用地市场。推进农村空心房整治，探索宅基地使用权流转，引导农村住宅集中建设。

切实抓好社会治理机制改革。强化信息化支撑。将“数字永州”建设作为社会治理改革的主抓手，以数字城管为突破口，推进电子政务、公共服务、社会治理三位一体的数字化网络平台建设，实现“数字化管理”。着力建好公民、法人、城市地理空间、城市部件、经济信息、房产房屋等六大类数据库。深化电子政务应用，加快电子政务交换、协同办公系统、政府在线服务工程建设。逐步建设数字乡村。强化网格化管理。积极推进新型社区建设，逐步将城市管理、人口管理、民政、社会保障、物业、卫生、治安、信访、党建等功能纳入网格中，实行小区域、模块式的扁平化管理，让社会治理落实到小区、到楼栋、到个人。各县区要进行网格化管理试点，中心城区全面推行网格化管理。以乡镇为区域试点建立综治工作站、综治小区和综治小组，实行三级网格化管理。强化全程化服务。整合基层社会事务管理和民生服务资源，联合办公，集中受理，实现“一个门进来，一站式服务，一揽子解决”。壮大社区力量，将街道和社区干部、城管人员、社保人员、片区民警、协管员、物业、保安等人员纳入网格管理员队伍。建立社会管理综合信息平台和指挥处置中心。

在抓好三项改革的同时，加快财税体制、投融资、招投标、国有林场、医疗卫生等方面的改革。有效控制和化解地方政府性债务风险，加强平台公司建设。积极探索合理设置乡村规模，研究重点乡镇发展建设的体制机制。

6、以主攻关键环节为重点，加快推进全面小康建设

壮大县域经济。把推进县域经济发展作为全面小康建设的重中之重，通过改革创新、项目支撑、政策扶持，加快完善有利于县域经济发展的体制机制。把项目建设放在第一位，加快建成一批强基础、增财力、惠民生的大项目好项目。实施工业强县战略，培育壮大一批骨干龙头企业，加快形成一批特色主导产业。大力发展非公有制经济，关心爱护企业家。全面放开建制镇和小城市落户限制，增强城镇产业发展、公共服务、吸纳就业、人口聚集功能。推动城乡互动融合发展，统筹推进小城镇建设、新农村建设和新型社区建设。加快公共服务、社会事业向农村延伸，促进城乡公共服务均等化。充分尊重农民和基层首创精神，增强农村发展活力，逐步缩小城乡差距，促进城乡共同繁荣。

强化扶贫攻坚。把打好扶贫开发攻坚战作为全面小康建设的关键环节，促进贫困地区早日实现全面小康。全面落实国家和省里扶贫开发政策，加大新田、江华两个国扶县和宁远、双牌、江永三个省扶县扶贫攻坚力度。推进精细化、精准化扶贫，重点抓好发展生产、健全公共服务和教育三件大事，实行一乡一策、一村一策，做到一户一本台账、一个脱贫计划、一套帮扶措施，全年新减少贫困人口13万人。强化产业扶贫，支持贫困地区资源合理开发利用，完善特色优势产业扶持政策。统筹保障贫困地区基本公共服务，坚持整村推进与重点帮扶相结合，促进农村最低生活保障制度与扶贫开发政策有效衔接，持续推动水、电、路、气、房和环境整治“六到户”。抓好贫困地区教育工作，实施“雨露计划”，加强农村劳动力技能培训，不能让贫困地区年轻人输在起跑线上。加大对贫困地区的信贷投入，努力满足扶贫对象发展生产的资金需求。创新扶贫工作机制，开展党政领导定点扶贫，引导社会资金投入扶贫开发。积极向上申报争取设立国家级南岭山区连片特困地区。

夯实基层基础。把强化基层基础工作作为全面小康建设的重要保障，高度重视基层建设，真情关心基层干部。积极发展村级集体经济，不断提高乡村运转保障水平，改善乡村工作条件。大力培养一批优秀致富带头人，增强引领发展、服务群众的能力。

7、以保障和改善民生为重点，加快推进和谐社会建设

强化民生保障。坚持就业优先，切实抓好高校毕业生、被征地农民、零就业家庭等的就业工作，全年新增城镇就业5．5万人，农村劳动力转移就业6．5万人，高校毕业生就业率稳定在85％以上，城镇零就业家庭动态援助率达到100％。扩大社会保险覆盖面，完成五险参保619万人次。稳步提高社会救助水平，强化低收入家庭和特困群体的基本生活保障，抓好应急救灾和防灾减灾工作，确保市福利中心、军供站等项目年内建成。创建一批省级示范敬老院和省级和谐文明示范社区。

发展社会事业。以教育均衡发展为目标，以名校建设为引领，以扩大投入为保障，以强化管理为支撑，着力打造人民群众更加满意、更加放心的优质教育，全年义务教育二类县区巩固率达到98％以上，三类县区达到97％以上，新增城区中小学学位1．5万个，其中中心城区5000个，建设合格学校60所、农村公办幼儿园25所，加快4所特殊教育学校建设。推进名校名师名校长建设，力争2—3所重点中学进入全省名校行列。开展名校托管农村中学、名师下乡支教活动。健全家庭经济困难学生资助体系，促进教育公平。积极发展民办教育，加大职业教育资源整合力度，支持湖南科技学院省市共建，促进各级各类教育全面发展。推动科技创新，实施省级以上科技计划项目45个以上，新认定高新技术企业5家以上，申请专利1500件以上，授权700件以上。继续深化医药卫生体制改革，加快卫生事业发展，扩大国家基本药物制度实施范围，稳步推进县级公立医院综合改革，促进基本公共卫生服务均等化，做好重大疾病防治工作，新农合参合率稳定在98％以上。稳定低生育水平，提高出生人口素质。继续实施文化惠民工程，抓好蘋洲文化广场、凤凰影视城等项目建设，培育一批龙头文化企业。加强社会主义核心价值体系建设，全面提高公民道德素质和社会文明程度。发展竞技体育，开展全民健身。认真落实各项民族政策，促进民族地区加快发展。完成村“两委”换届工作。抓好妇女、儿童工作。积极发展老龄事业，关爱“空巢”家庭，创建“百岁健康市”。进一步完善残疾人社会保障和服务体系建设。

加强生态建设。坚守生态底线，划定生态红线，既要金山银山，更要绿水青山，努力建设“天蓝、地绿、水净、景美、民富”的绿色新永州。按照资源和环境承载能力，划定保护区、限制开发区和优化开发区，全年要完成畜禽禁养区、限养区、适养区的划定。全面启动环保隐患大排查和大气污染防治行动，组织开展湘江流域综合治理、城乡环境治理和矿区治理，切实加强小锰矿、小造纸、小矿山、小冶炼关停整治。把好监管关、排放关、环评关，严格实行环保“三同时”制度和责任追究制度。积极申报国家重点生态功能区。加大植树造林力度，全年完成营造林120万亩，中心城区完成20万株大苗种植任务。

办好六件实事。一是抓好棚户区改造。全年实施改造项目56个、20895户。二是抓好城市小区管理。已建成的小区要完善公共设施配套，在建和拟建的小区要确保公共设施、物业管理和社会管理同步跟进。三是抓好农村环境污染治理。加大农村改水改厕力度，推进农村垃圾无害化处理，启动乡镇生活污水处理设施建设，解决畜禽养殖业污染问题，严控农业面源污染，减少工业污染。四是抓好放心食用油和米粉。加强生产加工、流通销售和餐饮消费监管，杜绝地沟油、泔水油、餐厨废弃油和劣质米粉进入餐饮行业。五是抓好“五小”水利建设。全年投资3．5亿元，完成8560处。六是抓好社会安全。狠抓“三级战略防控圈”和“七张网”建设，坚持警力下沉，增加街面见警率，深入开展专项整治行动，始终保持对犯罪分子的高压态势，实现防控网络全覆盖。建立健全安全生产责任体系，强化重点行业领域专项整治，有效防范和遏制重特大安全事故发生。

统筹推进国防和后备力量建设，健全国防动员机构机制，推进民兵工作调整转型，优化后备力量规模、结构和布局，不断提高遂行多样化军事任务的能力。强化全民国防教育，广泛开展军民共建和“双带双促”活动，促进军民融合深度发展。依法规范兵役工作，提高兵役工作质量。

8、以务实高效为重点，加快推进政府自身建设

依法治市，提升公信力。推进民主法制建设，全面落实市委的决策部署，坚决执行市人大及其常委会的决议、决定，自觉接受市人大及其常委会的法律监督和工作监督，支持和保障市政协履行政治协商、民主监督、参政议政职能，认真听取社会各界和新闻媒体的意见、建议。坚持科学民主依法决策，积极推行权力清单制度，大力推进决策公开、管理公开、服务公开、结果公开，做到阳光行政，建设透明政府。强化诚信意识，开展诚信教育，大力推进政府决策信用、行政信用、服务信用建设，建立健全失信责任追究机制，推动全社会形成重诺守信的良好氛围。

改革攻坚，提升创新力。始终把改革创新作为关键之举，把攻坚克难作为制胜之道，努力让政府工作富有创新、更有成效。要用长远的眼光谋划发展，既要认清当前的困难问题，更要看到未来的发展空间，坚定信心、发挥优势，抢抓机遇、加快发展。要用改革的思路推动工作，抓住全面深化改革的有利时机，加快推进体制机制建设，不断释放改革创新活力。要用创新的手段破解难题，打破传统观念，突破思维定势，敢于先行先试，勇于开拓创新，走出一条符合永州实际、富有时代特色的发展新路。

尽职履责，提升执行力。强化责任意识，始终把加快永州发展、增进人民福祉作为政府工作的首要之职、应尽之责，恪尽职守，勇于担当，集中精力谋大事、干实事，凝聚力量抓推进、促落实。强化责任分工，建立健全横向到边、纵向到底的工作责任体系，严格实行分级负责、层层负责、各负其责，对重大事项、重点项目逐一明确时间表、路线图、责任人，一项一项定责任，一环一环抓落实。严格责任追究，进一步完善目标管理责任制度、政府绩效评估制度、定期督促检查制度，对工作不到位、责任不落实，造成不良影响的，要严厉问责。

廉洁从政，提升形象力。增强廉洁从政意识，严格落实党风廉政建设责任制，深入推进惩治和预防腐败体系建设，加强对工程建设、产权交易、政府采购、专项资金管理使用等重点领域的监管，强化行政监察和审计监督，加强对领导干部任期经济责任审计和政府投资项目审计，认真开展群众反映强烈突出问题的专项治理，严肃查处各类违纪违法行为。认真执行党政机关厉行节约反对浪费条例，扎实开展党的群众路线教育实践活动，坚决抵制“四风”，切实做到干部清正、政府清廉。

各位代表，改革新潮催骏马，潇湘大地起宏图。承载光荣使命，肩负人民重托，让我们高举中国特色社会主义伟大旗帜，在市委的坚强领导下，同心同德，奋发进取，全面开启跨越赶超新征程，奋力谱写富民强市新篇章！
